# My Experiences in War—A Contrast, 1885-1900

**Published:** 1902-08

**Authors:** G. Sterling Ryerson

**Affiliations:** C. A. M. S.; Toronto, Ont.; Knight of the Order of St. John of Jerusalem in England


### BUFFALO MEDICAL JOURNAL.

VOL. XLII.—LVIII. AUGUST, 1902. No. 1.

### ORIGINAL COMMUNICATIONS.

### My Experiences in War—a Contrast, 1885-1900.[1]

BY LIEUT.-COL. G. STERLING RYERSON, M. D., C. A. M. S., Toronto, Ont.
Knight of the Order of St. John of Jerusalem in England.

I WISH to say how much I appreciate the honor done me by your association in inviting me to be present and to take part in this most interesting meeting. I do not know of an occasion when medical officers whose experience covers so long a period of time from 1861 to 1902, and so wide a sphere of action, the Philippines, South Africa, Cuba and the war of the Rebellion, have been gathered together to compare notes and to deduce from the sum of their experiences something which may be for the public benefit. I am not one of those who believe that the time is at hand when there shall be no more war and that the best efforts of humanity will be devoted to the arts of peace. I am rather of the opinion of my late friend Surgeon General Hamilton, who thought that so long as man had a cerebellum he would fight.

Wars are more frequent and more bloody at the end of the 19th than in the 1st century. Education and religion have not eradicated nor even lessened man's love of war. Indeed, the conditions of the every-day struggle for existence, is of the nature of war. Thomas Jefferson said that the world needs a little blood letting every twenty years, and history shows that there has been a war of more or less magnitude at these intervals of time.

To the medical profession falls the honor of mitigating the horrors of war and of minimising the ills and suffering of those who fight for their country. Our work of mercy knows neither race, creed nor color. A broad humanity covers them all.

I have been asked to say something about the medical side

1. Read at the 27th annual meeting of the Alumni Association, Buffalo University Medical College, May 2, 1902.

of the war in South Africa, but I cannot do so to my own satisfaction without relating briefly some of my experiences in the Northwest rebellion in Canada in 1885, especially as the two campaigns present many interesting points of contrast and it will add another campaign to the records of today's discussions.

On the night of March 27, 1885, I was roused from a comfortable slumber by the ringing of my telephone. Imagine my surprise when the commanding officer of my regiment ordered me to parade at 8 a.m. next day with my ambulance corps, prepared to leave for the northwest, where a rebellion of half-breeds and Indians had broken out. The rest of the night was spent in preparation and in hunting up my men. Our departure was postponed two days to enable arrangements for transport, and the like, to be made. On the morning of the 30th we left Toronto for the front. On April 1 we reached Ricotasing, the then end of the Canadian Pacific Railway's tracks, on the north shore of Lake [Huron. A long gap of forty-two miles had now to be crossed in open sleighs during the night with a temperature of twenty degrees below zero. The snow was from four to five feet deep, the track through the forest narrow and any deviation from the beaten path meant an upset in the dark. After an all night drive, we arrived nearly perished with cold, at Camp Desolation. We camped in the snow at this place until April 3, without tents or cover save our blankets. One man went stark mad, removed all his clothing and would have leaped into the fire had he not been prevented. On this date we embarked in open flat cars running on rails laid on the snow, which gave a serpentine movement to the train.

We ran 150 miles in this way, arriving at Port Munro late in the evening. On Easter Sunday, April 5, we marched 20 miles across the ice to McKellar's bay, then again took "palace" flat cars for 12 miles to Jack Fish Bay, where the night was passed; next day we marched 22 miles to Winston, through snow and slush, then more "palace" cars to Nepigon, reaching there at 10 p.m. There still intervened 14 miles between the ends of the track. It was intensely dark, cold and raining. All around was the gloomy primeval forest; between us and comfort lay a stretch of ice covered to the depth of a foot or more with slush and water. Plunging, struggling along arm in arm, the regiment advanced. Hour after hour the weary struggle proceeded and day was breaking when the head of the column debouched on terra firma again. Exhausted, the men threw themselves on the seats of cars and fell asleep instantly. The details of this march have never been published fully until now, and I think you will agree with me that it was a wonderful performance, especially when it is considered that the men were fresh from the counting house and shop and factory, without any preliminary training for the field.

You will ask what were the physical effects of such a trial of endurance? They were less serious than you might expect. Three men broke down by the revival of old rheumatic affections, one man lost his toes with frost-bite, one became acutely insane, but eventually recovered, one had orchitis secondary to gonorrhea, one accidental fracture of the ulna, one accidental gunshot wound of the thigh, a dozen or more snow blind from the terrible glare on the march across the ice in daylight. A small casualty bill considering the circumstances. The gunshot wound was interesting. A correspondent was showing his self-cocking revolver to an officer when it went off. The ball entered the thigh close to the outer side of the femoral artery, ran upward toward the abdomen for about five inches, when it abruptly turned backward and was lost in the loose tissue at the back of the thigh, where it still lies. I ascribe our successful march partly to the food, which consisted of fat pork, bread, butter and biscuits, enabling the men to withstand the cold. No rum ration was issued. Alcohol means death to men benumbed with cold.

My experience leads me to believe that total abstinence in active service under hardship is a factor for good,—in very hot and very cold climates at any rate. We traveled by train to Qu'Appelle station, 2,150 miles from Toronto, where our march of 250 miles across the prairie began. When the alkali plains were reached the men, who could not be prevented from drinking the water, suffered severely from diarrhea, which was controlled with difficulty.

On April 24 was fought the action at Fish Creek, my first experience of Indian fighting. The half-breeds and Indians were driven off with a loss on our side of 8 killed and 40 wounded. That night it rained, snowed and froze, so that the outposts and sentries had a hard time of it, though strange to say very few came into hospital as a result. My Northwest and South African experiences lead me to believe that Canadians are as hardy as army mules, and that they can kick quite as hard on occasions. The engagement at Batoche took place from May

9 to 12, at which our loss was 8 killed and 46 wounded. This brings me to a consideration of the wounds inflicted.

The weapons used by the enemy were Winchesters, Sniders and smooth bore guns with a few Sharpe rifles. The Winchester produces a small wound of entrance and a huge wound of exit. The Snider, a large entrance and a huge exit. When bone was hit it usually splintered and fractured in all directions; an exception was the case of a man who was shot through the condyles of the femur at short range. A clean hole was bored without apparent involvement of the joint or shaft of the bone. He made a good recovery. Where joints were entered there was extensive destruction of parts necessitating resection or amputation.

Joints into which fractures entered became permanently anchylosed. I observed that even slight wounds were slow of healing. A tough scar formed which separated very slowly,—a great contrast to the Mauser wounds. Of pyemia there was only one case, that of a man who refused to have his leg amputated for a penetrating wound of the knee-joint. The primary dressings were crude, the first aid packet not being in use at that time. Multiple wounds were unknown owing to the slow firing of those days compared with the present. Wounds of the brain were all fatal. One man carried a round two ounce ball in his head for a week when he suddenly died, probably from secondary hemorrhage. All who were shot through the abdomen died—contrast the penetrating wounds of this region by the Mauser bullet—as did also those suffering from penetrating wounds of the chest. Slugs and round balls bruise and lacerate, but if not at very close range run around bone.

A curious wound is produced by two round balls flattened on one side; there is one wound of entrance and two of exit, often very remote one from the other, so one is puzzled to know where is the other wound of entrance. Secondary hemorrhage is common, owing to the laceration and bruising of vessels which do not give way at once.

To continue my narrative, we advanced by boat or by trail until we reached Fort Pitt, whence expeditions were sent out in chase of Big Bear. With his capture ended this trying little war, fought entirely by Canadian Volunteer soldiers. It will give some idea of the vastness of that northern land when I say that when we turned toward home we had 3,500 miles to travel, and that we were ten days coming down the Saskatchawan river by boat, traveling twenty hours a day, for there is little night in those northern latitudes in summer.

### SOUTH AFRICA.

I come now to the second part of this paper, my experiences in South Africa. On the 21st of January, 1900, I embarked for South Africa, on the troop ship Laurentian, with "D" and "E" Batteries, Royal Canadian Artillery, and on the 29th day from sailing, landed at Capetown.

We landed in a cape "southeaster;" fine, sharp dust was blowing in all directions. It was intensely hot, temperature, 105° in the shade. Along the docks lay transports three deep. On shore, negroes, malays, Chinamen, Colonials, English Tommies, and Highlanders jostled and swore in their respective tongues. Horses, mules, oxen, steam traction engines added to the noise and confusion. Mountains of food and war-like stores encumbered the dock. Above, the sweet blue sky of South Africa, in the distance, the towering heights of Table Mountain with its table cloth of cloud. What a contrast to the silence, the gloom of the forest, and the bitter cold of the north shore of my former experience!

Three days later I left for the front in a comfortable saloon carriage,—another contrast. How curious a land is South Africa? A land so often described, but yet indescribable. A vast area of plain studded with natural fortresses, called kopjies; traversed by great chains of mountains and wide rivers, which today are dry and tomorrow are rushing torrents, which none may cross, a land without herbage or trees, yet of a fertile soil, needing only water to render it most prolific of vegetation, as may be seen about Capetown and in southern Cape Colony. Through the vast area meanders a little narrow guage railway, so narrow that horses cannot stand athwart the little cattle tracks, but must stand sideways or with twisted necks and endure the torment of a long confinement and the want of water and proper sidings for disembarking them. Is it any wonder that they suffered and died? The road runs slowly upward for a 1,000 miles until a level of 6,500 feet above the sea is reached. Great ravines and dry water courses intersect the country, which must be spanned with bridges, and these were blown up by the enemy as they retired before our forces. On this toy railway all supplies of food, munitions of war, men, guns, horses, mules, forage, hospital equipment had to be carried for the army. The task was not an easy one.

After two days and nights of travel, I arrived at Orange River, then the immediate base of supplies for Lord Roberts's army. A few thousand men guarded the great stores of food and warlike supplies, but a large portion of the camp area was covered by a general hospital of 500 beds, under the command of Lt.-Col. Battersby, R.A.M.C. The patients were most comfortably disposed in galvanised iron huts, ingeniously constructed so as to be easily unscrewed and removed. Comfortable beds and bedding were provided, a fair number of female nurses were on duty and the much abused hospital orderly was much in evidence. The experience of this war has confirmed the experience of private life,—that men do not make good nurses. They have not the ready sympathy nor patient unselfish devotion to duty that women possess, and I believe that in the future the number of army nurses will be largely increased and men retained for the heavier work of lifting patients, and the like. The pay of the army nurse should be increased in the British service,—it is too ridiculously small. In Canada a nurse ranks as a lieutenant and has corresponding pay and authority over the men employed in the wards.

A few days later I pushed on to Modder River of sad memory, a shallow trench of muddy water bordered with bushes, a river which was a little later to play so large a rôle in the production of enteric fever. To the south, lay a plain as level as a billiard table with no more shelter for marksman than was afforded by a few crumbling ant heaps. Behind the river were kopjies of considerable height. A couple of miles away to the left arose the belting crags of Magersfontein. Here I first saw Mr. Johnnie Boer, as a prisoner of war. Tall, swarthy, rugged, unkempt, he is a typical frontiersman. I have seen many of his kind in the great West. I pushed on to Kimberley, where I arrived a week after the relief of the siege. It was surprising to notice how little damage had been done to the town by the bombardment. For weeks 100 pound shells fell in the place at regular intervals all day, yet little damage was done. Everyone I met had hairbreadth escapes to relate and pieces of shell to show, which might have killed them but did not. Food was scarce and the principal item of diet was soup of unknown composition. Of wheaten bread there was none, but a heavy black kind, made of rye flour and bran was abundant.

There were comparatively few sick in the town, but soon the sick and wounded began to arrive in large numbers from Paardeberg, where 1,000 came in one day. Under this pressure the drill hall, Masonic Temple, public schools and other buildings were converted into hospitals, and every effort was made to meet the requirements of the situation. The medical staff worked night and day, and as at Bloemfontein a few weeks later, did all that men could do and, as usual with our profession, got little thanks for it.

The cause of the epidemic is to be found in the consumption of the Modder River water, there being no other to be had, fouled by the enemy imprisoned at Paardeberg drift by Lord Roberts's army, and filled with the carcasses of dead men and animals. This led to the great epidemic at Bloemfontein, a month later. Owing to the pressure on the narrow guage railway, of which I have already spoken, it was impossible to get a full equipment of hospital supplies either to Kimberley or Bloemfontein.

I had now the opportunity of studying the wounds inflicted by the Mauser bullet, the Lee-Metford, and artillery fire. I cannot give you the latest statistics of the percentages of death from wounds, but it is very small as compared with former wars. Up to the end of 1900, 12,637 officers and men had been wounded, of these only 732 had died. Men shot through the chest had been on duty again in four weeks, men shot through the abdomen were at work in five weeks or less, in six weeks men shot through the knee joint were able to walk about without assistance. Nothing like it had ever been seen in war before. I attribute these results to the aseptic character of the bullet, its high velocity, to the prompt application of the first aid dressing and to the eminently efficient treatment which the wounded received at the hands of the medical officers. The Mauser bullet has justly been described as a merciful one. Its action, however, upon human tissues, depends upon the range at which it is fired. It has been noticed that when it is fired at short ranges, within 200 yards, it has an explosive action. The nickel case seems to expand and become detached, causing a severe lacerated and contused wound, which heals but slowly. If it strikes at this range it crushes and destroys it. At longer ranges it makes a clean drill hole in bone and if it strikes soft parts only, a very small wound is made, there being little difference between the wound on entry and that of exit, with but little bleeding, unless a large vessel is injured.

In the case of the soft nosed or dum-dum bullet the injury is severe, the wound of entry is small but that of exit is very large; great masses of tissue are torn off with much contusion. When it strikes bone, the bullet mushrooms and pulverises and disintegrates it. If the range is very long, 2,000 feet or more, the soft nose bullet mushrooms and causes an extensive wound notwithstanding the range. It has been alleged that explosive bullets were used by the Boers. I have never seen a case which would justify this statement, nor have I ever seen an explosive bullet, but I have seen Mauser cartridges, which were split at regular intervals, evidently in process of manufacture, and I have seen them with the tips filed off.

As to the so-called poison bullets they were simply green with verdigris, the action of the grease in which they are usually dipped to preserve them from damp. This would rub off in the barrel when fired. Besides Mausers, the Boers used thousands of Martini-Henry rifles, which, as you know, throws a heavy chronicle ball similar to the Snider. The wound produced is extensive. You will recollect my statements relative to the Snider.

I had the opportunity at Kimberley, Bloemfontein and elsewhere of examining considerable numbers of Boer wounded and of observing the action of the Lee-Metford bullet. It inflicts a wound very similar in character to that of the Mauser, though the bullet is somewhat heavier and larger. The effect of shellfire is interesting. To begin with the smallest, the "pom-pom." I have known a shell of this class to go right through a man's face from front to rear, leaving a large hole, without exploding. Generally when they strike bone they blow a man all to pieces. They very seldom hit directly, but a man may be filled with small fragments. The noise they make in exploding is terrifying to the most hardened soldier. Common shell fly into a number of fragments which tear and lacerate frightfully, generally necessitating amputation. Shrapnel shell, contain 100 round bullets, which spread out fan-shape when the projectle bursts, and are usually deadly when they strike, but I once saw a Boer shot through the stomach from side to side by one of these missiles without seeming to be much inconvenienced by it. Lydite has not been found as effective in land fighting as was expected. If the shells fall on soft ground they do not explode, when they hit a rock the concussion and explosion is terrific and destroys everthing within reach. The fumes cause severe headache, which the Boers say is counteracted by a few drops of vinegar.

Compared with my experience in the Northwest campaign Mauser wounds are innocuous. For instance, I have known a man to be shot through the knee-joint and walk in four weeks. He rejoined his regiment and in the next action was shot through the same joint again, with the same result. Penetrating wounds of the chest, unless they opened a large vessel, generally were followed by recovery. The experience of penetrating wounds of the abdomen is that they are immediately fatal if a large vessel is wounded, but wounds of the viscera are almost harmless. They are best left alone as resections have not been followed by good results. I have observed that perforations of the stomach heal readily if the viscus happens to be empty at the time, and the soldier refrains from drinking water. After two or three months discomfort is often complained of, caused possibly by adhesions which have formed. It will be interesting to learn the ultimate result of these wounds. A case of perforating wound of the liver, which came under my notice had rather hard luck, the patient was brought about forty miles in an ambulance and when seen was in a state of collapse. He recovered from his wound and was taken down with enteric fever; from this also he recovered, when he was attacked with dysentery from which he succumbed.

Wounds of the brain and its membranes are, of course, generally fatal, but I saw one recovery from very extensive destruction of the calvarium with contusion of the brain, followed by sloughing. He developed epileptic symptoms. I have heard of lodged Mauser bullets in the brain with recovery, but I have not seen a case. I have, however, seen an extensive laceration of the frontal lobe with loss of the eye make a good recovery, and I have seen both eyes shot out by a bullet passing transversely behind, and one in which the optic nerves were divided without much injury to the eyeballs.

I have thus far related some of my surgical experiences which form the dramatic side of war. Let me now say a few words about the medical aspect, which after all is the more important, for statistics prove that in war while 5 per cent. die from wounds or are killed forthwith, 15 per cent. die of disease, and the South African war is no exception to the rule.

Enteric or typhoid fever is the great scourge of armies in temperate climates to which in hot ones dysentery and cholera are added. I heard of no case of cholera in South Africa, but in East Indian campaigns it is a prominent feature. It is a sort of "bull" to say that prevention is the great remedy, yet it is so. The sanitation of armies leaves much to be desired. Every army should have a chief sanitary officer with a large staff under him who should have full power to act peremptorialy. It is generally a quartermaster's work, a layman who knows nothing or next to nothing of his duties, or does not attend to them at all and men die like flies. Contaminated water was largely responsible for the epidemics in South Africa, but a large factor was the exposure of food for the troops to the action of myriads of flies which probably came straight from a feast on the saliva of enterics. The latrines were open and alive with flies. Urine passed on the sand dried and was blown hither and thither. The same can be said of solid excreta. Rigid sanitary arrangements would save thousands of lives in war as in peace.

In conclusion, allow me to say a few words about the use and abuse of the Red Cross. Under present conditions with long range arms of precision, the arm band worn by the individual is useless. It cannot be seen. At long range ambulances with their white covers cannot be distinguished from transport or amunition wagons. The cross on the white field is so small it cannot be discerned. The Boers made crosses the whole size of the wagon, which was better. Colonel Neilson, D.G.M.S., Canadian Army, makes a suggestion which I think is a good one—namely, that all connected with the medical service on the field of battle should wear red and that the tilts of the ambulance wagons should be painted red. All nations now have a field service dress according to the climate in which war is being carried on. The British red coat and the American blue will never be seen again on active service. Is it not a good idea to make the life-saving service as conspicuous as possible and thus avoid the regretable "Red Cross incidents" which are a constant source of contention?

I cannot too warmly state my appreciation of the honor you have done me in asking me to be present today and I wish the University of Buffalo God speed on her mission,—enlightenment and progress.

60 College Street.

